# Supplementary material for: Sperm membrane proteins DCST1 and DCST2 are required for sperm-egg interaction in mice and fish
Source: Commun Biol. 2022 Apr 7;5:332. doi: 10.1038/s42003-022-03289-w (PMC8989947; doi:10.1038/s42003-022-03289-w)
Supplement: Supplementary file 2 — Supplementary information [file 42003_2022_3289_MOESM2_ESM.pdf]

Supplementary information

Phylogenetic tree of the DC-STAMP-like containing proteins and analysis of DCST1/2 protein conformation.

Sequences were collected in an HMM search using the PFAM DC-STAMP model against NCBI-nr protein or UniProt reference proteomes databases applying highly significant E-value thresholds (<1e-10), selected for a wide taxonomic range, and aligned with mafft (-linsi, v7.427)[1-3]. A region conserved within DCST1, DCST2, DCSTAMP and OCSTAMP orthologues (covering Homo sapiens DCST1 46-599) was extracted with Jalview[4]. A maximum-likelihood phylogenetic tree was reconstructed with IQ-TREE version 2.1.3 using the "Q.plant+I+G4" model selected by ModelFinder and branch support obtained with the ultrafast bootstrap method v2[5-7]. The visualization was done with iTOL v6[8]. Branches supported by ultrafast bootstrap values (>=95%) were marked with a blue dot. Sequence accessions were added next to the species names.

The alignment of mouse and zebrafish DCST1 and DCST2 amino acid sequences was performed with Clustal Omega (https://www.ebi.ac.uk/Tools/msa/clustalo/) and visualized with JalView (https://www.jalview.org/)[4]. The DC-STAMP-like protein domain prediction was derived from pfam protein domains (http://pfam.xfam.org/). Transmembrane helices were predicted with TMHMM (http://www.cbs.dtu.dk/services/TMHMM/) and Phobius (https://phobius.sbc.su.se/)[9,10].

Generation of *Dcst1* and *Dcst2* mutant mice.

Dcst1 and *Dcst2* mutant mice were generated using 4 guide RNAs (gRNAs) (**Table S2**) as described previously[11-13]. Genotyping PCR was conducted with primer sets (**Table S2**) and KOD-Fx neo. The PCR condition was 94 °C for 3 minutes, denaturing at 94°C for 30 seconds, annealing at 55°C (for *Dcst2* indel mutants) or 65°C (for *Dcst1* indel and *Dcst2* deletion mutants) for 30 seconds, and elongation 72°C for 30 seconds for 35 or 40 cycles in total, followed by 72°C for 2 minutes.

Generation of Tg mice.

Sequences of *Dcst1* cDNA-3xHA tag and *Dcst2* cDNA-3xHA with a rabbit poly A signal were inserted under mouse *Clgn* promoter. The linearized DNA was injected into the pronucleus of zygotes, and the injected eggs were transferred into the ampulla of pseudopregnant females. Genotyping PCR was conducted with primer sets (**Table S3**) and KOD-Fx neo. The PCR condition was 94°C for 3 minutes, denaturing at 94°C for 30 seconds, annealing at 65°C for 30 seconds, and elongation 72°C for 1 minute (for *Dcst1*-3xHA), and 2 minutes (for *Dcst2*-3xHA) for 35 cycles in total, followed by 72°C for 2

minutes.

Quantitative PCR (qPCR) to analyze *Dcst1* and *Dcst2* expression levels

The synthesized cDNA (5 ng), primer sets (**Table S4**), and THUNDERBIRD Next SYBR qPCR Mix (TOYOBO) were used for qPCR. The condition for qPCR was 95°C for 30 seconds, denaturing at 95°C for 5 seconds, annealing at 65°C for 10 seconds for 40 cycles in total. For melting curve, the samples was treated at 95°C for 15 seconds, followed by increasing the temperature by 0.3°C from 60°C. *Actb* was used as a reference gene, and the relative difference in the expression level was calculated by the ΔΔCt method.

Morphology and histological analysis of a mouse testis.

The testicular weight and body weight of $Dcst1^{d1/wt}$, $Dcst1^{d1/d1}$, $Dcst2^{d25/wt}$, and $Dcst2^{d25/25}$ males (8-24 weeks old) were measured. The testis was fixed with Bouin's fluid (Polysciences) at 4°C overnight. Fixed samples were dehydrated by increasing the ethanol concentration, and then embedded with paraffin. Paraffin sections (5 μm) were stained with 1% periodic acid solution (Wako) for 10 minutes. Then, these samples were counterstained with Mayer hematoxylin solution for 3 to 5 minutes, dehydrated in increasing ethanol concentrations, and finally mounted in Entellan new (Merck).

CRISPR/Cas9-mediated zebrafish mutant generation.

Homozygous mutants for *dcst1*, *dcst2*, and *dcst1/2* were generated in zebrafish using CRISPR/Cas9-mediated mutagenesis through the use of single gRNAs (sgRNAs) generated as previously described[14]. SgRNAs targeting exons 2 and 3 for *dcst1* or exon 4 for *dcst2* (**Table S5**) were co-injected with Cas9 protein into one-cell TLAB embryos to generate the single knock-out mutants (**Figure S12B**). $Dcst1/2^{-/-}$ zebrafish were generated in the same manner, but by injecting sgRNAs targeting exons 2 and 3 for *dcst1* in conjunction with those targeting exon 4 for *dcst2* into a *dcst1* mutant background (149 bp deletion, 14 bp insertion) to increase the chances of mutagenesis for both genes on the same allele. For all mutants, injected embryos were grown to adulthood and out-crossed to *wt* TLAB zebrafish; the offspring were then screened by PCR (**Table S5**) for heterozygous mutations in *dcst1*, *dcst2*, or both loci to identify founders. Siblings of fish found to carry mutations in the *dcst1* or *dcst2* locus were grown to adulthood and in-crossed to generate homozygous mutant fish. Amplicon sequencing of adult fin-clips revealed the different mutations as a 2-bp substitution combined with a 50-bp insertion and 1-bp substitution in exon 2, and 4-bp deletion in exon 3 of $dcst1^{-/-}$; a 7-bp deletion in exon-4 for $dcst2^{-/-}$; and a 155-bp deletion in *dcst1* exon 3 combined with a 4-bp insertion

followed by a 64-bp insertion in *dcst2* exon 4 in case of the double mutant *dcst1/2*$^{-/-}$. Genotyping of *dcst1*$^{-/-}$, *dcst2*$^{-/-}$, and *dcst1/2*$^{-/-}$ mutant fish was performed using PCR (**Table S5**). Detection of mutations was performed using standard agarose gel electrophoresis (wt amplicon for *dcst1*: 311 bp, wt amplicon for *dcst2*: 394 bp, *dcst1*$^{-/-}$ amplicon: 358 bp; *dcst2*$^{-/-}$ amplicon: 387 bp; amplicon sizes in the *dcst1/2*$^{-/-}$ double mutant: *dcst1*$^{-/-}$ amplicon: 156 bp; *dcst2*$^{-/-}$ amplicon 467 bp). To confirm the presence of a truncated mRNA product for both mutants, cDNA was generated using the iSCRIPT cDNA Synthesis Kit (BioRad) from RNA isolated from mutant testis tissue. Sanger sequencing using primers within the open reading frame for both *dcst1* and *dcst2* was performed using the primers listed below (**Table S5**). Confirming that the mutations lead to a truncated mRNA product, the *dcst1*$^{-/-}$ cDNA encoded only 15 amino acids with a premature termination codon, compared to 676 amino acids for wt, the *dcst2*$^{-/-}$ cDNA encoded 183 amino acids (709 amino acids in wt), and the *dcst1/2*$^{-/-}$ cDNA encoded 25 amino acids for *dcst1* and 170 amino acids for *dcst2* (**Figure S12C**). The recovered mutant alleles were registered with ZFIN as vbc14, vbc15, and vbc16 respectively.

Generation of Dcst2 RING finger domain constructs.

To clone the coding sequence of the Dcst2 RING finger domain, RNA was isolated from adult testis using the standard TRIzol (Invitrogen) protocol followed by phenol/chloroform extraction. cDNA was synthesized using the iSCRIPT cDNA Synthesis Kit (BioRad) and served as template for the amplification of the sequence underlying Dcst2 (566-709) (Dcst2-RING_F and Dcst2-RING_R). The PCR product was introduced into BamHI/EcoRI-cut *pMTB-actb2:MCS-sfGFP* (a derivative of *pMTB-actb2:H2B-Cerulean*, a kind gift from Sean Megason) by Gibson assembly[15] to generate an in-frame fusion to sfGFP. The resulting ORF is flanked by a SP6 promoter and SV40 3’ UTR.

mRNA injection of zebrafish embryos.

TLAB embryos were collected immediately after being laid and dechorionated with pronase (1 mg/ml). Dechorionated one-cell stage embryos were injected with 100 pg mRNA and cultured at 28°C in E3 medium (5 mM NaCl, 0.17 mM KCl, 0.33 mM $CaCl_2$, 0.33 mM $MgSO_4$, 10^{-5}% Methylene Blue). mRNA encoding Dcst2(566-709)-sfGFP was prepared by *in vitro* transcription using the mMESSAGE mMACHINE SP6 kit (Invitrogen) from a plasmid containing the SP6 promoter and SV40 3’ UTR. Shield-stage (6 hpf) embryos were fixed with 3.7% formaldehyde at 4°C overnight. Immunofluorescence staining of embryos was performed as was done for zebrafish sperm

immunocytochemistry, but with goat anti-mouse IgG Alexa Fluor 594 as secondary antibody. The embryos were imaged in PBS in a watch glass with a stereomicroscope (Lumar, Zeiss) with the 0.8x Neo Lumar S objective and 5x zoom (40x magnification).

Zebrafish adult tissue RNA-Seq.

Adult zebrafish tissues (muscle, spleen, liver, intestine, heart, brain, fin, skin, testis) were dissected from adult *wt* (TLAB) zebrafish (three biological replicates for each sample). Total RNA was isolated using the standard TRIzol protocol and assessed for quality and quantity based on analysis on the Fragment Analyzer. PolyA+ RNA was selected with the poly(A) RNA Selection Kit (LEXOGEN), following the manufacturer's instructions. Stranded cDNA libraries were generated using NEBNext Ultra Directional RNA Library Prep Kit for Illumina (New England Biolabs) and indexed with NEBNext Multiplex Oligos for Illumina (Dual Index Primer Set I) (New England Biolabs). Library quality was checked on the Fragment Analyzer and sequenced on a Illumina Hiseq 2500 with the SR100 mode. RNA-seq reads were processed according to standard bioinformatic procedures. Reads were mapped to Ensembl 102 gene models (downloaded 2021.01.25) and the GRCz11 Danio rerio genome assembly, with Hisat2 v2.1.0[16] using the Ensembl transcriptome release 102. A custom file was generated by adding bouncer based on its position coordinates [exon = chr18:50975023-50975623 (+ strand); CDS = chr18:50975045-50975422 (+ strand)]. Quantification at the gene level (transcript per million (TPM)) was performed using Kallisto (v0.46.0)[17]. The RNA-seq data set was deposited to Gene Expression Omnibus (GEO) and is available under GEO acquisition number GSE171906. RNA-seq data of ovary and oocyte-stage samples have been published previously and are available under GEO acquisition numbers GSE111882 (testis, ovary, mature oocytes)[18] and GSE147112 (oogenesis, mature oocytes)[19].

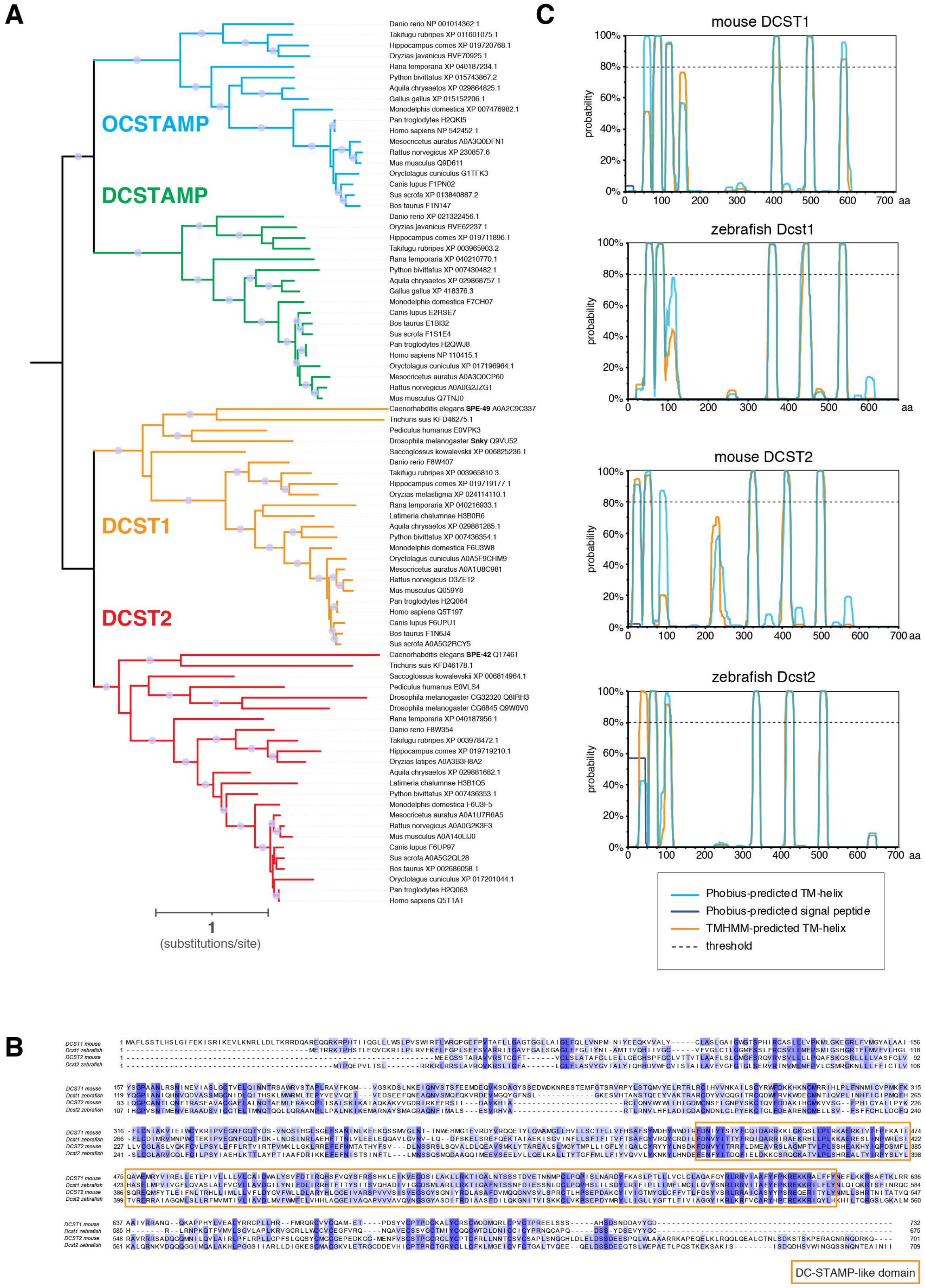
A
OCSTAMP
DCSTAMP
DCST1
DCST2
Danio rerio NP 001014362.1
Takifugu rubripes XP 011601075.1
Hippocampus comes XP 019720768.1
Oryzias javanicus RVE70925.1
Rana temporaria XP 040187234.1
Python bivittatus XP 015743867.2
Aquila chrysaetos XP 029864825.1
Gallus gallus XP 015152206.1
Monodelphis domestica XP 007476982.1
Pan troglodytes H2QKI5
Homo sapiens NP 542452.1
Mesocricetus auratus A0A3Q0DFN1
Rattus norvegicus XP 230857.6
Mus musculus Q9D611
Oryctolagus cuniculus G1TFK3
Canis lupus F1PN02
Sus scrofa XP 013840887.2
Bos taurus F1N147
Danio rerio XP 021322456.1
Oryzias javanicus RVE62237.1
Hippocampus comes XP 019711896.1
Takifugu rubripes XP 003965903.2
Rana temporaria XP 040210770.1
Python bivittatus XP 007430482.1
Aquila chrysaetos XP 029868757.1
Gallus gallus XP 418376.3
Monodelphis domestica F7CH07
Canis lupus E2RSE7
Bos taurus E1BI32
Sus scrofa F1S1E4
Pan troglodytes H2QWJ8
Homo sapiens NP 110415.1
Oryctolagus cuniculus XP 017196964.1
Mesocricetus auratus A0A3Q0CP60
Rattus norvegicus A0A0G2JZG1
Mus musculus Q7TNJ0
Caenorhabditis elegans SPE-49 A0A2C9C337
Trichuris suis KFD46275.1
Pediculus humanus E0VPK3
Drosophila melanogaster Snky Q9VU52
Saccoglossus kowalevskii XP 006825236.1
Danio rerio F8W407
Takifugu rubripes XP 003965810.3
Hippocampus comes XP 019719177.1
Oryzias melastigma XP 024114110.1
Rana temporaria XP 040216933.1
Latimeria chalumnae H3B0R6
Aquila chrysaetos XP 029881285.1
Python bivittatus XP 007436354.1
Monodelphis domestica F6U3W8
Oryctolagus cuniculus A0A5F9CHM9
Mesocricetus auratus A0A1U8C981
Rattus norvegicus D3ZE12
Mus musculus Q059Y8
Pan troglodytes H2Q064
Homo sapiens Q5T197
Canis lupus F6UPU1
Bos taurus F1N6J4
Sus scrofa A0A5G2RCY5
Caenorhabditis elegans SPE-42 Q17461
Trichuris suis KFD46178.1
Saccoglossus kowalevskii XP 006814964.1
Pediculus humanus E0VLS4
Drosophila melanogaster CG32320 Q8IRH3
Drosophila melanogaster CG6845 Q9W0V0
Rana temporaria XP 040187956.1
Danio rerio F8W354
Takifugu rubripes XP 003978472.1
Hippocampus comes XP 019719210.1
Oryzias latipes A0A3B3H8A2
Aquila chrysaetos XP 029881682.1
Latimeria chalumnae H3B1Q5
Python bivittatus XP 007436353.1
Monodelphis domestica F6U3F5
Mesocricetus auratus A0A1U7R6A5
Rattus norvegicus A0A0G2K3F3
Mus musculus A0A140LIJ0
Canis lupus F6UP97
Sus scrofa A0A5G2QL28
Bos taurus XP 002686058.1
Oryctolagus cuniculus XP 017201044.1
Pan troglodytes H2Q063
Homo sapiens Q5T1A1
1
(substitutions/site)
C
mouse DCST1
zebrafish Dcst1
mouse DCST2
zebrafish Dcst2
probability
aa
Phobius-predicted TM-helix
Phobius-predicted signal peptide
TMHMM-predicted TM-helix
threshold
B
DCST1 mouse
Dcst1 zebrafish
DCST2 mouse
Dcst2 zebrafish
DC-STAMP-like domain

Figure S1. Conservation of DCST1 and DCST2 across bilaterians and predicted protein structure.

A) Phylogenetic tree of the DC-STAMP-like domain containing proteins. A maximum-likelihood phylogenetic tree revealed an early split between DCSTAMP (green) and OCSTAMP (blue) proteins on the one hand and DCST1 (orange) and DCST2 (red) on the other hand. Common protein names for *C. elegans* SPE-42/SPE-49 and *D. melanogaster* Sneaky are included. The branch lengths represent the number of substitutions per site.

B) Amino acid sequence alignment between mouse and zebrafish DCST1 and DCST2. Letters with blue shading indicate the percentage of sequence identity (dark blue: 100% identity). The DC-STAMP-like protein domain is highlighted in an orange box.

C) Predicted transmembrane helices for mouse and zebrafish DCST1 and DCST2. Plots show predicted probabilities for transmembrane helices [TMHMM (orange) and Phobius (blue)] in mouse and zebrafish DCST1 and DCST2.

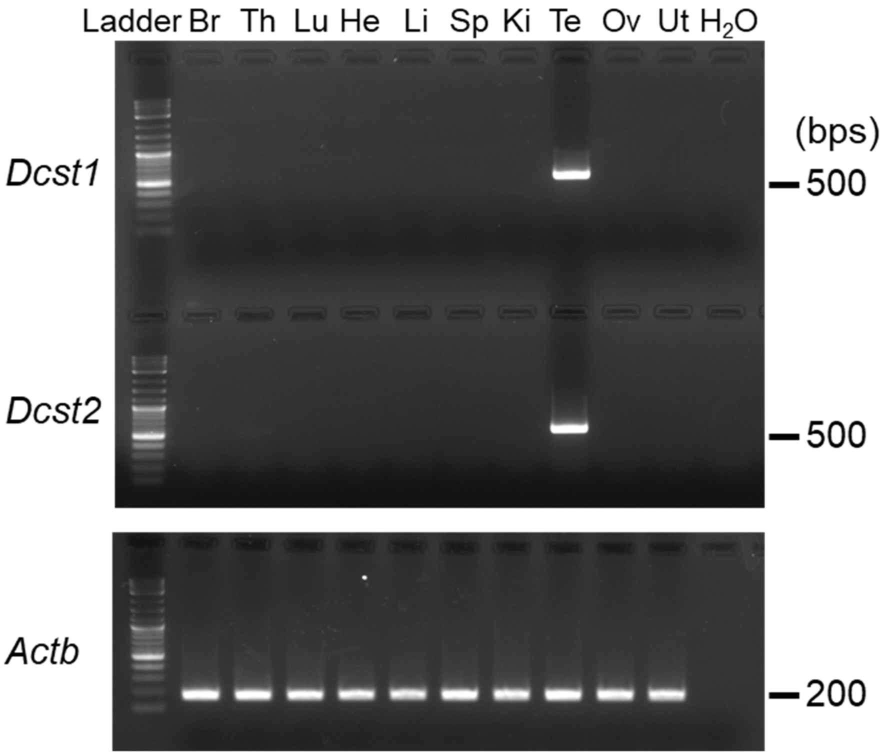

Figure S2. Multi-tissue gene expression analysis. *Dcst1* and *Dcst2* are abundantly expressed in the mouse testis. Beta actin (*Actb*) was used as the loading control. The uncropped and unedited images in Figure 1A were shown. Br, brain; Th, thymus; Lu, lung; He, heart; Li, liver; Sp, spleen; Ki, kidney; Te, testis; Ov, ovary; Ut, uterus.

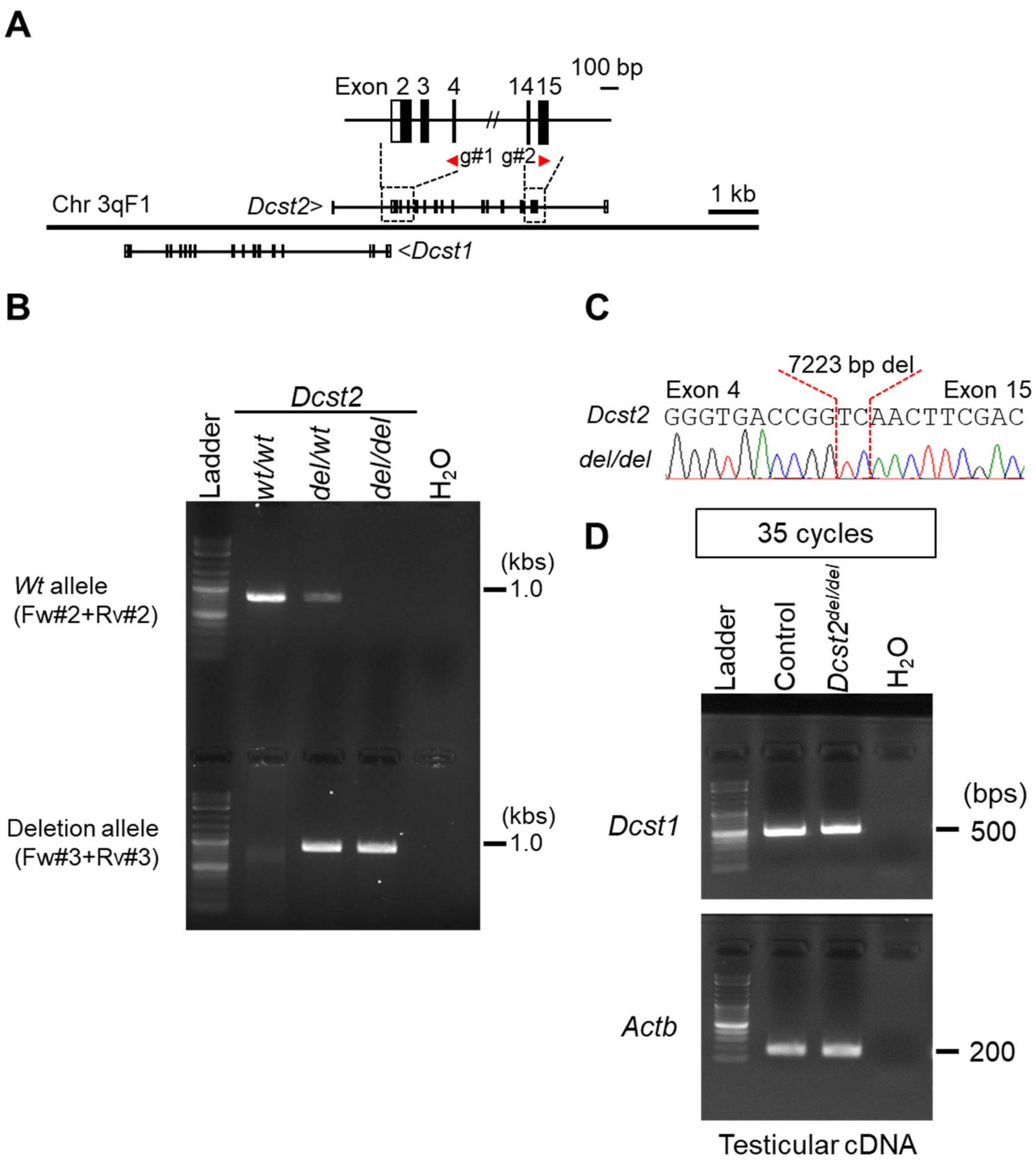

Figure S3. Generation of *Dcst2*$^{del/del}$ mice.

A) gRNA design. Mouse *Dcst1* and *Dcst2* are adjacent genes in a head-to-head arrangement such that parts of their 5' genomic regions overlap. To delete the coding region of *Dcst2*, we designed 2 gRNAs in exon4 and 15 of *Dcst2*. Black colored boxes show the coding region.

B and C) Genotyping with PCR and direct sequencing. Four primers were used for the genotyping PCR. The amplicons were subjected to direct sequencing, and the mutant allele has a 7223 bp deletion and 2 bp insertion.

D) Detection of *Dcst1* mRNA in *Dcst2*$^{del/del}$ testis.

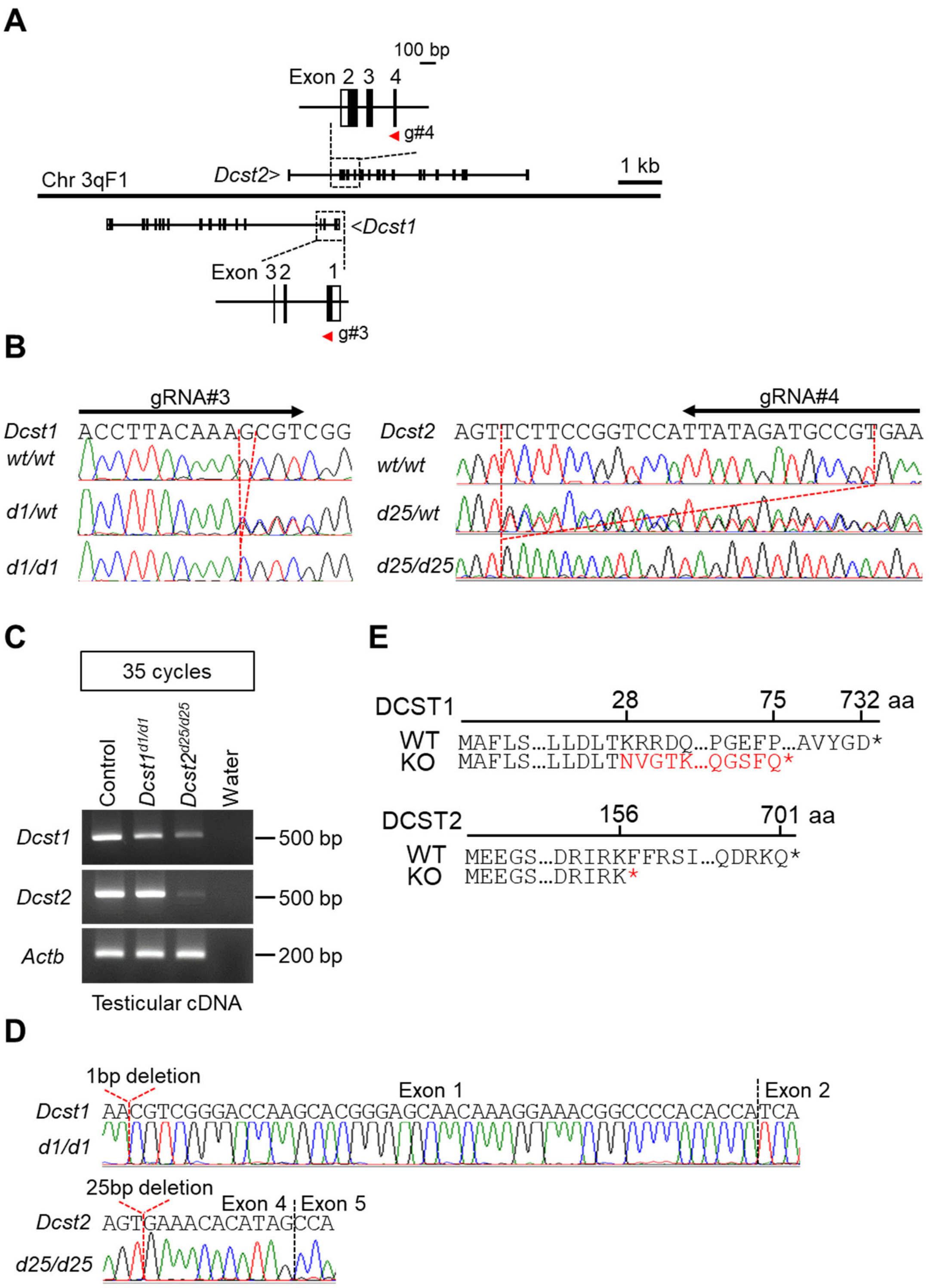
A
100 bp
Exon 2 3 4
g#4
Dcst2>
1 kb
Chr 3qF1
<Dcst1
Exon 3 2 1
g#3
B
gRNA#3
Dcst1 ACCTTACAAAGCGTCGG
wt/wt
d1/wt
d1/d1
gRNA#4
Dcst2 AGTTCTTCCGGTCCATTATAGATGCCGTGAA
wt/wt
d25/wt
d25/d25
C
35 cycles
Control
Dcst1 d1/d1
Dcst2 d25/d25
Water
Dcst1
Dcst2
Actb
500 bp
500 bp
200 bp
Testicular cDNA
D
1bp deletion
Exon 1
Exon 2
Dcst1 AACGTCGGGACCAAGCACGGGAGCAACAAAGGAAACGGCCCCACACCATCA
d1/d1
25bp deletion
Exon 4
Exon 5
Dcst2 AGTGAAACACATAGCCA
d25/d25
E
DCST1
28
75
732 aa
WT MAFLS…LLDLTKRRDQ…PGEFP…AVYGD*
KO MAFLS…LLDLTNVGTK…QGSFQ*
DCST2
156
701 aa
WT MEEGS…DRIRKFFRSI…QDRKQ*
KO MEEGS…DRIRK*

Figure S4. Generation of *Dcst1*$^{d1/d1}$ and *Dcst2*$^{d25/d25}$ mice.

A) gRNA design. To generate indel mutant mice of *Dcst1* and *Dcst2*, we designed 2 gRNAs in exon1 of *Dcst1* and exon 4 of *Dcst2*. Black colored boxes show the coding region.

B) Genome sequence of *Dcst1* and *Dcst2* in *Dcst1*$^{d1/d1}$ and *Dcst2*$^{d25/d25}$ mice. The mutant alleles of *Dcst1* and *Dcst2* have a 1-bp deletion in *Dcst1* and a 25-bp deletion in *Dcst2*, respectively.

C and D) Detection and cDNA sequencing of *Dcst1* and *Dcst2* mRNAs in *Dcst1*$^{d1/d1}$ and *Dcst2*$^{d25/d25}$ testes. *Dcst1* mRNA in *Dcst1*$^{d1/d1}$ testis and *Dcst2* mRNA in *Dcst2*$^{d25/d25}$ testis were detected (panel C), but *Dcst1* and *Dcst2* cDNA sequencing have a 1-bp and 25-bp deletion in *Dcst1* and *Dcst2*, respectively.

E) Predicted amino acid sequences of *Dcst1/2* KO mice. The indel mutation causes the amino acid changes (red-colored letters) due to the frame shift, leading to the appearance of premature stop codon (*).

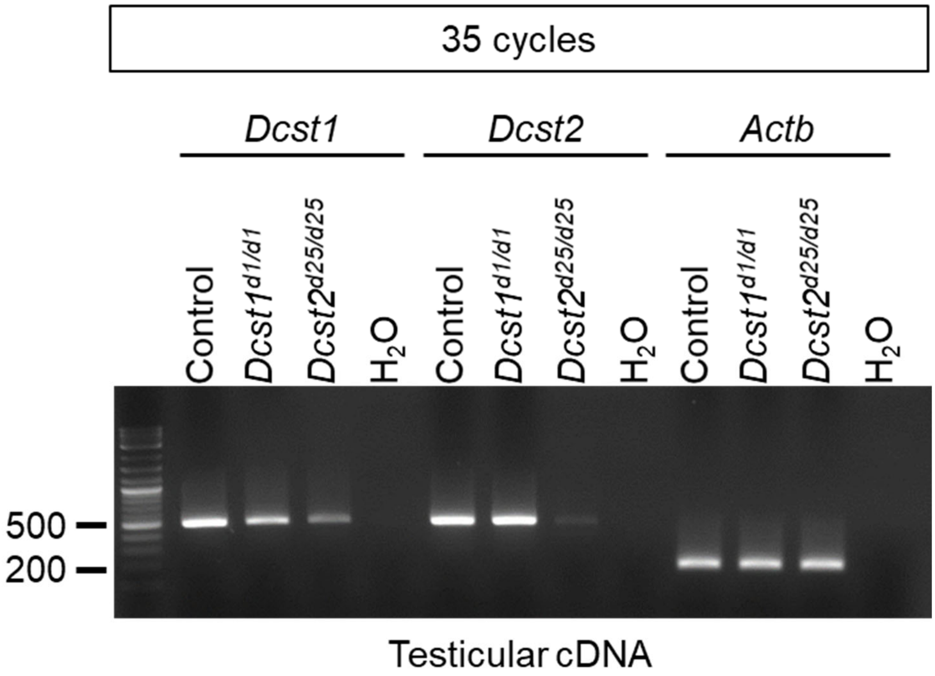

Figure S5. Detection of *Dcst1* and *Dcst2* mRNAs in *Dcst1*$^{d1/d1}$ and *Dcst2*$^{d25/d25}$ testes. *Dcst1* mRNA in *Dcst1*$^{d1/d1}$ testis and *Dcst2* mRNA in *Dcst2*$^{d25/d25}$ testis were detected. The uncropped and unedited images in Figure S4C were shown.

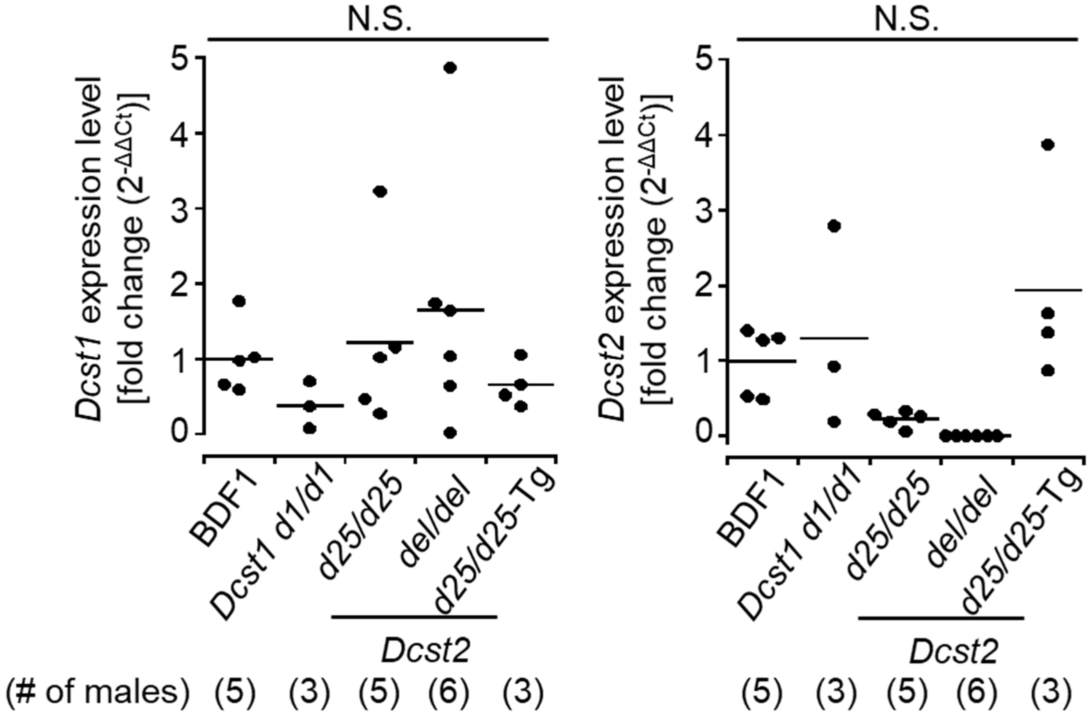

Figure S6. *Dcst1/2* expression levels in each mutant using quantitative PCR. *Actb* was used as a reference gene, and the relative difference in the expression level was calculated by the ΔΔCt method. N.S.: not significance.

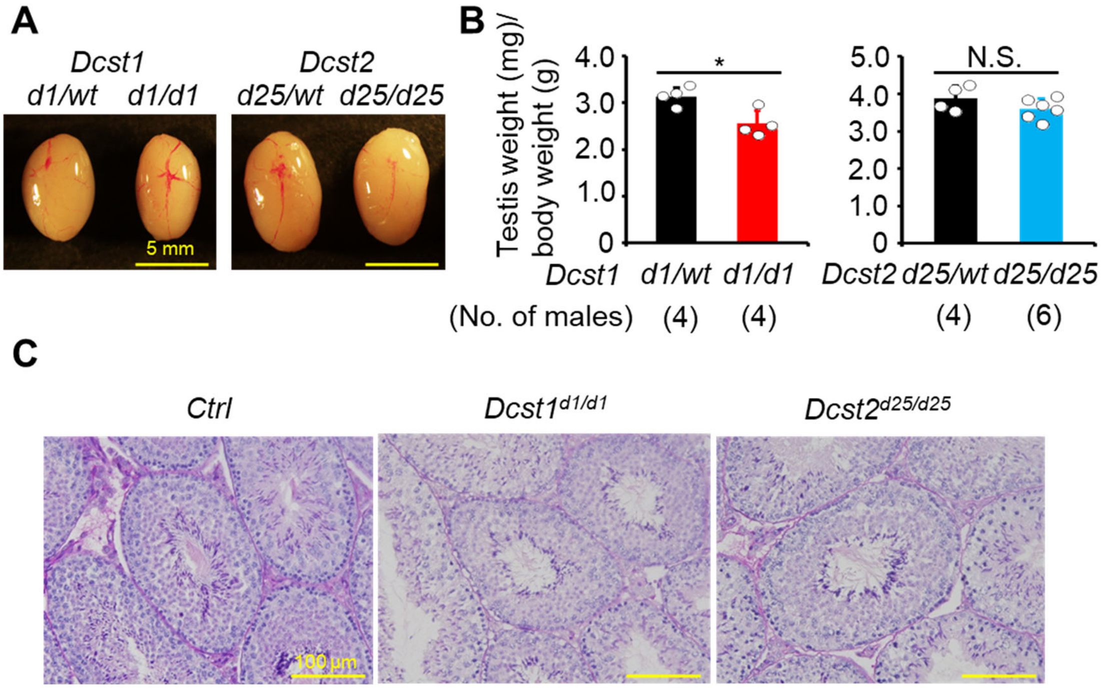

Figure S7. Analysis of *Dcst1*$^{d1/d1}$ and *Dcst2*$^{d25/d25}$ testes.
A) Testis morphology.
B) Testis weight (mg) / body weight (g). The value of *Dcst1*$^{d1/d1}$ testis slightly decreased compared with the *Dcst1*$^{d1/wt}$ testis. *: $p < 0.05$., N.S.: not significant ($p = 0.24$).
C) Histological analysis. There is no overt abnormality in *Dcst1*$^{d1/d1}$ and *Dcst2*$^{d25/d25}$ spermatogenesis. Shown are PAS-hematoxylin-stained sections of testes of wild-type (Ctrl), *Dcst1*$^{d1/d1}$ and *Dcst2*$^{d25/d25}$ mice.
All values are shown as the mean ± SD.

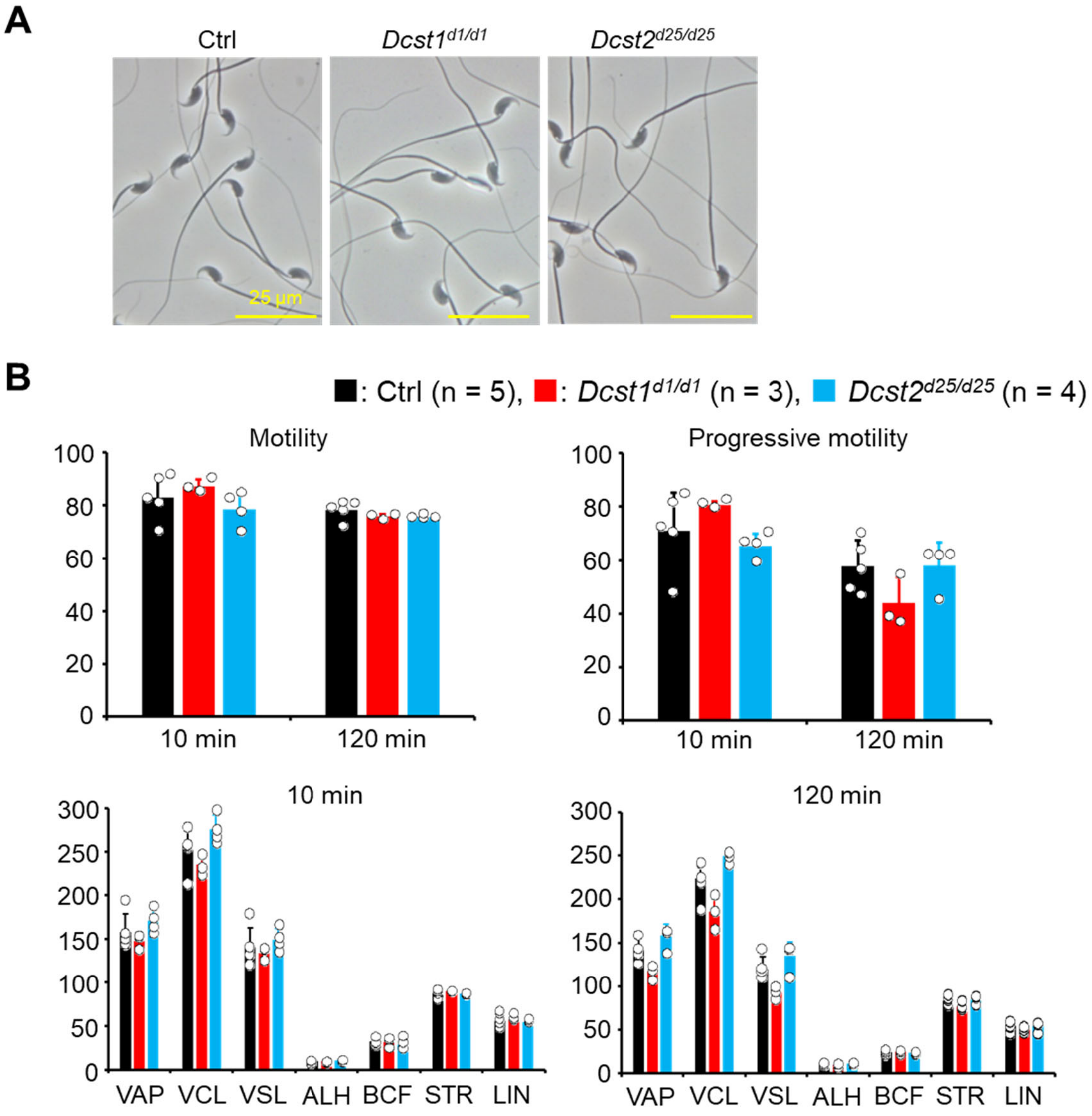

Figure S8. Analysis of *Dcst1* KO and *Dcst2* KO sperm.

A) Sperm morphology.

B) Sperm motility. There was no difference in sperm motility parameters between Ctrl, *Dcst1* KO and *Dcst2* KO sperm. Sperm from $Dcst1^{d1/wt}$ and $Dcst2^{d25/wt}$ males were used as the control. VAP: average path velocity, VSL: straight line velocity, VCL: curvilinear velocity, ALH: amplitude of lateral head, BCF: beat cross frequency, STR: straightness of trajectory, LIN: linearity.

All values are shown as the mean ± SD.

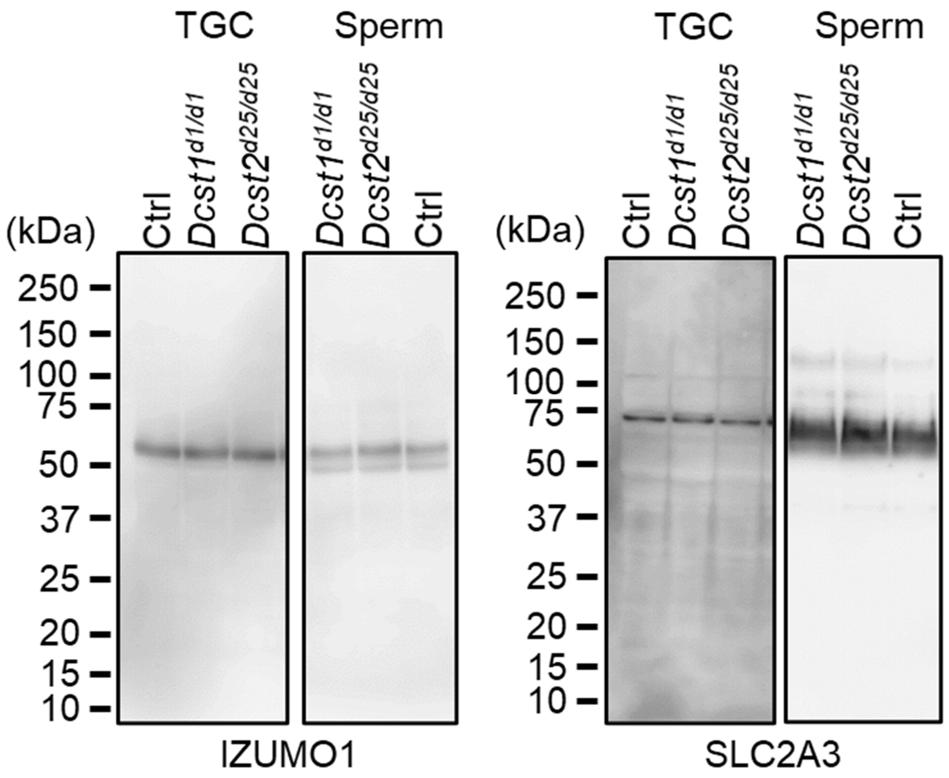

Figure S9. Detection of IZUMO1. The band signals of IZUMO1 in TGC and sperm of *Dcst1*$^{d1/d1}$ and *Dcst2*$^{d25/d25}$ male mice were comparable to the control wild-type sperm. SLC2A3, one of proteins in sperm tail, was used as the loading control. The uncropped and unedited images in Figure 2B were shown.

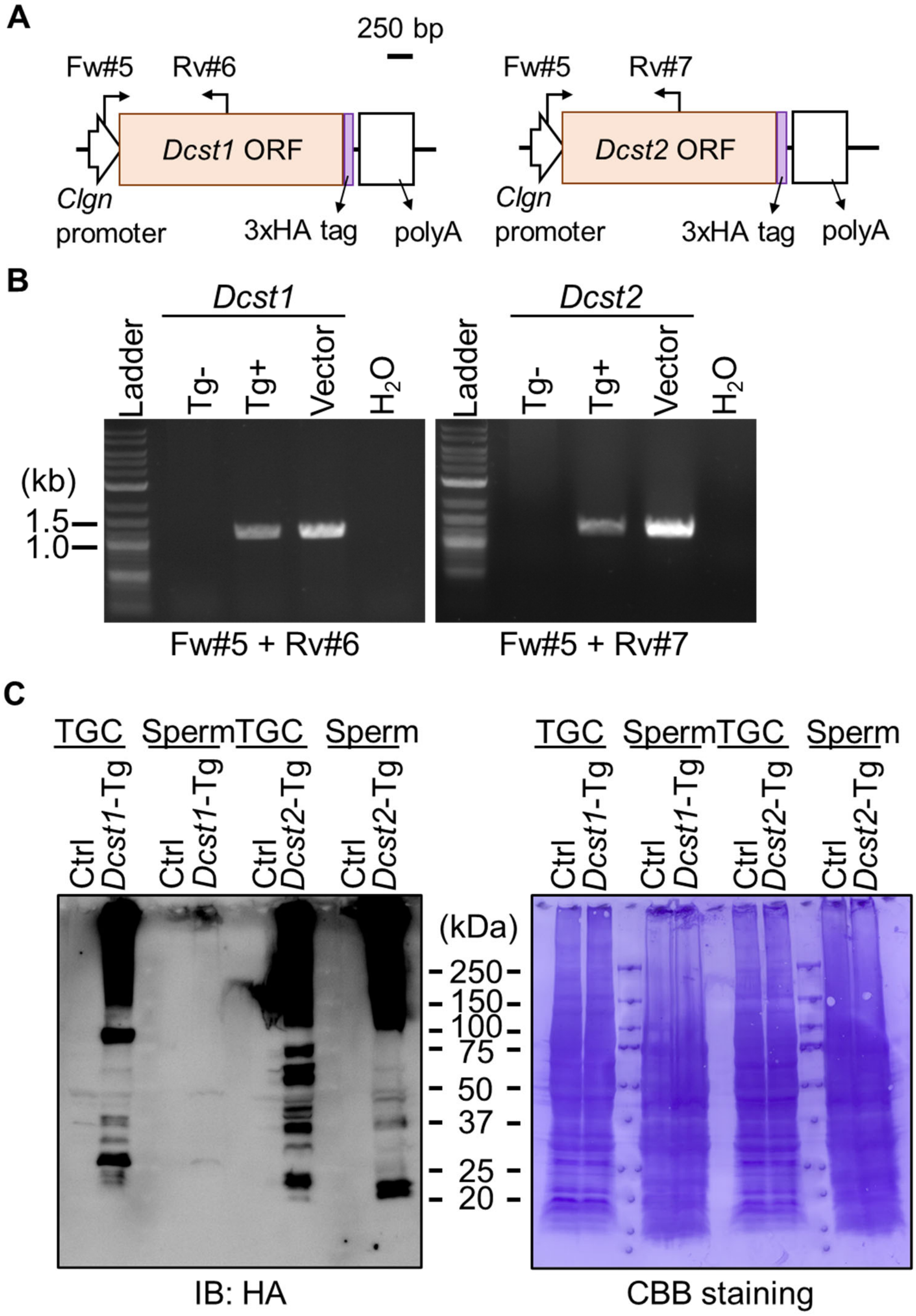

Figure S10. Generation of *Dcst1*-3xHA and *Dcst2*-3xHA Tg mice.

A) Schematic presentation of generating *Dcst1*-3xHA and *Dcst2*-3xHA Tg mice. The transgenes with ORF regions of mouse *Dcst1* and *Dcst2* and 3xHA tag were expressed as a fused protein under the testis-specific Calmegin (*Clgn*) promoter.

B) Genotyping of *Dcst1*-3xHA and *Dcst2*-3xHA Tg mice.

C) Detection of HA-tagged DCST1/2. TGC: testicular germ cells.

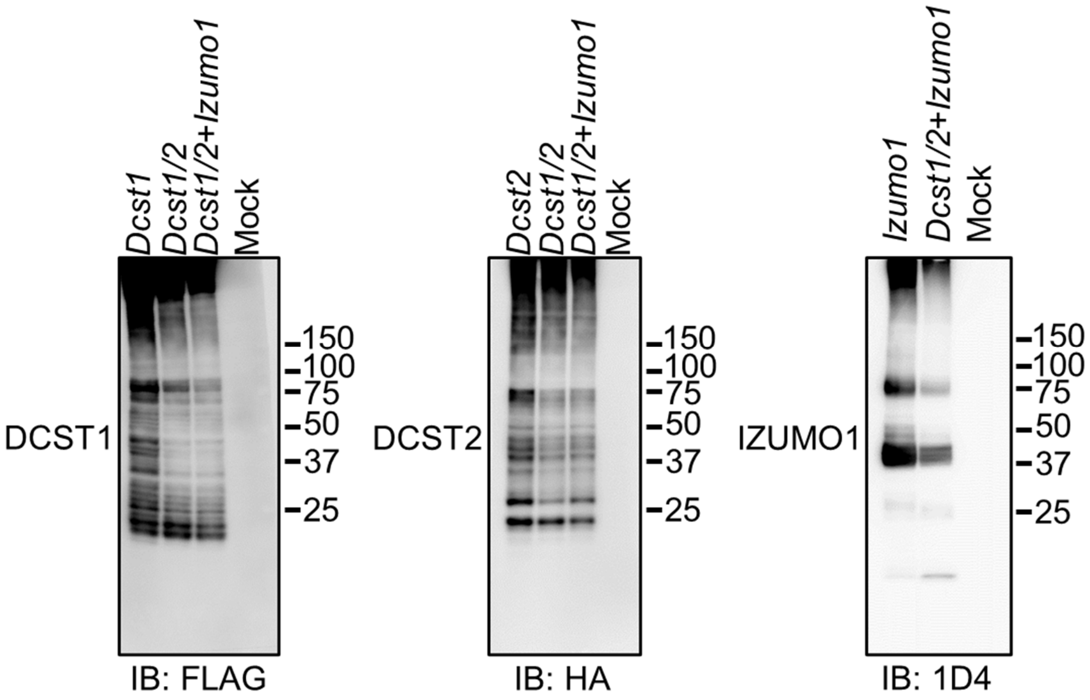

Figure S11. Detection of DCST1/2 and IZUMO1. FLAG-tagged DCST1, HA-tagged DCST2, and 1D4-tagged IZUMO1 were detected in HEK293T cells overexpressing *Dcst1*-3xFLAG, *Dcst2*-3xHA, and *Izumo1*-1D4. The uncropped and unedited images in Figure 4A were shown.

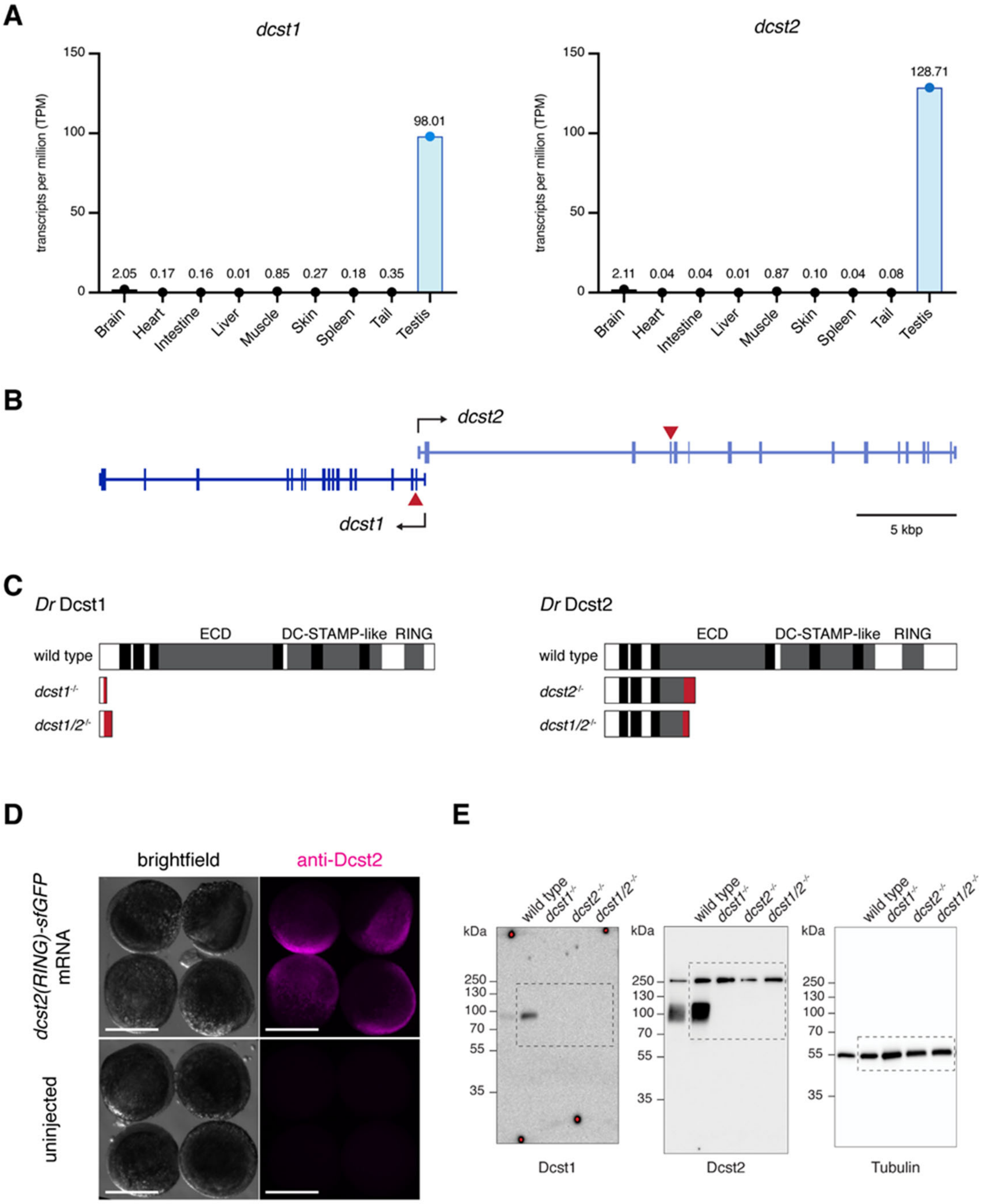
A
dcst1
transcripts per million (TPM)
150
100
50
0
2.05
0.17
0.16
0.01
0.85
0.27
0.18
0.35
98.01
Brain
Heart
Intestine
Liver
Muscle
Skin
Spleen
Tail
Testis
dcst2
2.11
0.04
0.04
0.01
0.87
0.10
0.04
0.08
128.71
B
dcst2
dcst1
5 kbp
C
Dr Dcst1
ECD
DC-STAMP-like
RING
wild type
dcst1-/-
dcst1/2-/-
Dr Dcst2
dcst2-/-
D
brightfield
anti-Dcst2
dcst2(RING)-sfGFP mRNA
uninjected
E
wild type
dcst1-/-
dcst2-/-
dcst1/2-/-
kDa
250
130
100
70
55
35
Dcst1
Dcst2
Tubulin

Figure S12: Expression of *dcst1* and *dcst2* in zebrafish.

A) *Dcst1* and *dcst2* genes are specifically expressed in adult testis in zebrafish. RNA-Seq analysis of *dcst1* and *dcst2* gene expression levels in various adult tissues. Amongst all the tissues tested, *dcst1* and *dcst2* transcripts are strongly enriched in adult testis. The y-axis shows TPM values (transcripts per million).

B) *Dcst1* and *dcst2* gene locus in zebrafish. *Dcst1* and *dcst2* genes overlap in their 5' ends. The red triangles indicate the sites of the introduced mutations in *dcst1* and *dcst2*.

C) Dcst1 and Dcst2 domain organization and mutant proteins. Zebrafish Dcst1 and Dcst2 are multi-pass transmembrane proteins. Predicted transmembrane domains (black, Phobius prediction[10]), the extracellular domain (ECD), the DC-STAMP-like domain (DC-STAMP) and C_4C_4 RING finger domain[20] are indicated. The *dcst1* and *dcst2* mutant alleles encode for truncated proteins. The aberrant translation caused by frameshift indels up to the premature termination codon is indicated in red.

D) The anti-Dcst2 antibody detects overexpressed Dcst2 protein in zebrafish embryos by immunofluorescence. Immunofluorescent detection of Dcst2 protein (magenta) in zebrafish embryos. Embryos were either injected at the 1-cell stage with 100 pg of *dcst2 (RING)*[20]*-sfGFP*) mRNA (top; overexpression of Dcst2 (RING)-sfGFP) or not injected (bottom; negative control). Embryos were fixed after 6 hours, followed by immuno-staining using an antibody recognizing the RING-domain of zebrafish Dcst2. Scale bar: 500 µm.

E) Detection of Dcst1 and Dcst2 in zebrafish sperm lysate. The uncropped and unedited images of Figure 5C are shown.

Supplementary Movie Legends

Movie S1. Egg observation after IVF using *Dcst1* KO sperm.

Movie S2. Egg observation after IVF using *Dcst2* KO sperm.

Movie S3. Wild-type sperm approach to micropyle. Wild-type sperm stained with Mitotracker Deep-Red was added to wild-type eggs and images were acquired following sperm addition.

Movie S4. *Dcst2* mutant sperm approach to micropyle. *Dcst2* mutant sperm stained with Mitotracker Deep-Red was added to wild-type eggs and images were acquired following sperm addition.

Movie S5. *Dcst2* mutant sperm are unable to stably bind to wild-type eggs. Time lapse of sperm binding assay with wild-type (left) and *dcst2*$^{-/-}$ (right) sperm stained with Mitotracker Deep Red and wild-type eggs. After 2 minutes following sperm addition, wild-type sperm are stably bound to the oolemma while *dcst2*$^{-/-}$ mutant sperm are unable to bind.

Supplemental Table S1. Primer sequences for the tissue expression analysis				
**Genes**	**Accession #**	**Forward (5'-3')**	**Reverse (5'-3')**	**Predicted size (bp)**
Dcst1	ENSMUSG00000042672	ATGGCGTTCCTCTCATCAAC	TTGGACCTTAGATTGGCCGC	500
Dcst2	ENSMUST00000208216	GGGCCTTGTGCGAACACCCT	ACGGGAGTGCGGATGACCGT	491
Actb	ENSMUSG00000029580	CATCCGTAAAGACCTCTATGCCAAC	ATGGAGCCACCGATCCACA	171

Supplemental Table S2. Primer sequences for the genotyping and gRNA sequences.						
**Genes**	**Accession #**	**gRNAs (5'-3')**	**Forward (5'-3')**	**Reverse (5'-3')**	**Total cycles**	**Predicted size wild/mutant (bp)**
Dcst1	ENSMUSG00000042672	TGCTGGACCTTACAAAGCGT (gRNA #3)	CAGCGTTGACTATGGCTGCTGGG (Fw #1)	GCCATCTCTCCAGCCCCAGG (Rv #1)	35	617/616
Dcst2	ENSMUSG00000109293	ACCGGAAGAACTTGCGTATC (gRNA #1)<br>TCGAAGAAAGGCCCCCGAGC (gRNA #2)	AACCAGAGCGTAGGTCGTGG (for wild allele, Fw #2)	GAGATATACGGGGTTGAGGG (for wild allele, Rv #2)	40	822/N.D.
			CACCTCAGCCTAACCATTGG (for deletion allele, Fw #3)	TGTGCCTAACAAACCCCAGG (for deletion allele, Rv #3)	40	8106/885
		GTGTTTCACGGCATCTATAA (gRNA #4)	TAAAGCTATTGCCCAGAAGG (for indel, Fw #4)	ACTTAGGATAATAAGATTGG (for indel, Rv #4)	40	147/122

N.D.: not detected.

Supplemental Table S3. Primer sequences for the genotyping of Tg mice.				
**Genes**	**Forward (5'-3')**	**Reverse (5'-3')**	**Total cycles**	**Predicted size (bp)**
Dcst1	TTGAGCGGGCCGCTTGCGCACTGG (Fw#5)	ATTGGCACACAGATCATGTTGTTGAAGAGTGGGAG (Rv#6)	35	1202
Dcst2	TTGAGCGGGCCGCTTGCGCACTGG (Fw#5)	ACATTGTCGAATTTGTCGGAGTTCAGATAATAGTA (Rv#7)	35	1301

Supplemental Table S4. Primer sequences for qPCR analysis				
**Genes**	**Accession #**	**Forward (5'-3')**	**Reverse (5'-3')**	**Predicted size (bp)**
Dcst1	ENSMUSG00000042672	GGCAGTGTTCAAGGGCATGG	GGTTCCGAACATCTCTGTGC	163
Dcst2	ENSMUST00000208216	AAGGTCGTTTCGGGATTCCC	TGCCAAGCAGTTTCATCACG	163
Actb	ENSMUSG00000029580	CATCCGTAAAGACCTCTATGCCAAC	ATGGAGCCACCGATCCACA	171

Supplemental Table S5. Primers for zebrafish		
**Oligo**	**Sequence**	**Comment**
*dcst1*_sgRNA_exon2	**TAATACGACTCACTATA**ggttaaacgcacttgaatggGTTTTAGAGCTAGAAATAGCAAG	**T7 5' sequence**, target sequence, 3' TRACER OLIGO ANNEALING SEQUENCE
*dcst1*_sgRNA_exon3	**TAATACGACTCACTATA**ggcacagaaaactctgacagGTTTTAGAGCTAGAAATAGCAAG	**T7 5' sequence**, target sequence, 3' TRACER OLIGO ANNEALING SEQUENCE
*dcst2*_sgRNA_exon4a	**TAATACGACTCACTATA**ggataagatcaaggaaatggcgGTTTTAGAGCTAGAAATAGCAAG	**T7 5' sequence**, target sequence, 3' TRACER OLIGO ANNEALING SEQUENCE
*dcst2*_sgRNA_exon4b	**TAATACGACTCACTATA**ggcgaggaatgcttactcgaGTTTTAGAGCTAGAAATAGCAAG	**T7 5' sequence**, target sequence, 3' TRACER OLIGO ANNEALING SEQUENCE
Tracer_oligo	AAAAGCACCGACTCGGTGCCACTTTTTCAAGTTGATAACGGACTAGCCTTATTTTAACTTGCTATTTCTAGCTCTAAAAC	Common oligo used to generate guide RNAs for zebrafish mutagenesis by annealing and *in vitro* transcription using T7[4]
*dcst1*_exon2_F	GTTTTCTTTAGCGGTGTAGC	Primers for genotyping zebrafish *dcst1* mutants, yield 311-bp wild-type product
*dcst1*_exon2_R	ACAACTGGTTTTGCAGTTAC	
*dcst2*_exon4_F	GTCAGCCATAATGTTGTGTG	Primers for genotyping zebrafish *dcst2* mutants, yield 394-bp wild-type product
*dcst2*_exon4_R	ACGTTTCTTAGAGTACGAGC	
*dcst1*_amp_F	CAGCAATGGAAACTAGACG	Primers used to amplify *dcst1* cDNA from *dcst1*$^{-/-}$ zebrafish testis
*dcst1*_amp_R	GGAGGTAAAGTTTATCAGCAGCC	
*dcst2*_amp_F	ATGACTCCACAGGAACCTG	Primers used to amplify *dcst2* cDNA from *dcst2*$^{-/-}$ zebrafish testis
*dcst2*_amp_R	GCCTTAGATTATGTTAATAGCCTCCG	
*dcst1*_seq_R	ATTATGCTGGATGTTCGCAA	Primer used to sequence *dcst1*$^{-/-}$ cDNA
*dcst2*_seq_F	CTTCATCTGTACCTTGGTGATC	Primers used to sequence *dcst2* cDNA
*dcst2*_seq_R	ACGTTTCTTAGAGTACGAGC	
dcst2-RING_F	gctacttgttctttttgcaggatcc*gccaccatg*AACAAGGTAGACCAGCAAC	to clone the CDS of Dcst2 (566-709) into BamHI/EcoRI *pMTB-actb2:MCS-sfGFP*
dcst2-RING_R	ACACTCCTGATCCTCCTGAgaattcgattatgttaatagcctccgtattc	

References

1 Eddy, S. R. Profile hidden Markov models. *Bioinformatics* **14**, 755-763 (1998).

2 El-Gebali, S. *et al.* The Pfam protein families database in 2019. *Nucleic Acids Res* **47**, D427-D432 (2019).

3 Katoh, K. & Toh, H. Recent developments in the MAFFT multiple sequence alignment program. *Brief Bioinform* **9**, 286-298 (2008).

4 Waterhouse, A. M., Procter, J. B., Martin, D. M., Clamp, M. & Barton, G. J. Jalview Version 2--a multiple sequence alignment editor and analysis workbench. *Bioinformatics* **25**, 1189-1191 (2009).

5 Minh, B. Q. *et al.* IQ-TREE 2: New Models and Efficient Methods for Phylogenetic Inference in the Genomic Era. *Mol Biol Evol* **37**, 1530-1534 (2020).

6 Kalyaanamoorthy, S., Minh, B. Q., Wong, T. K. F., von Haeseler, A. & Jermiin, L. S. ModelFinder: fast model selection for accurate phylogenetic estimates. *Nat Methods* **14**, 587-589 (2017).

7 Hoang, D. T., Chernomor, O., von Haeseler, A., Minh, B. Q. & Vinh, L. S. UFBoot2: Improving the Ultrafast Bootstrap Approximation. *Mol Biol Evol* **35**, 518-522 (2018).

8 Letunic, I. & Bork, P. Interactive Tree Of Life (iTOL): an online tool for phylogenetic tree display and annotation. *Bioinformatics* **23**, 127-128 (2007).

9 Krogh, A., Larsson, B., von Heijne, G. & Sonnhammer, E. L. Predicting transmembrane protein topology with a hidden Markov model: application to complete genomes. *J Mol Biol* **305**, 567-580 (2001).

10 Kall, L., Krogh, A. & Sonnhammer, E. L. Advantages of combined transmembrane topology and signal peptide prediction--the Phobius web server. *Nucleic Acids Res* **35**, W429-432 (2007).

11 Noda, T. *et al.* Sperm proteins SOF1, TMEM95, and SPACA6 are required for sperm-oocyte fusion in mice. *Proc Natl Acad Sci U S A* **117**, 11493-11502 (2020).

12 Noda, T., Oji, A. & Ikawa, M. Genome Editing in Mouse Zygotes and Embryonic Stem Cells by Introducing SgRNA/Cas9 Expressing Plasmids. *Methods Mol Biol* **1630**, 67-80 (2017).

13 Noda, T. *et al.* Nine genes abundantly expressed in the epididymis are not essential for male fecundity in mice. *Andrology* **7**, 644-653 (2019).

14 Gagnon, J. A. *et al.* Efficient mutagenesis by Cas9 protein-mediated oligonucleotide insertion and large-scale assessment of single-guide RNAs. *PLoS One* **9**, e98186 (2014).

15 Gibson, D. G. *et al.* Enzymatic assembly of DNA molecules up to several hundred

kilobases. *Nat Methods* **6**, 343-345 (2009).
16 Kim, D., Paggi, J. M., Park, C., Bennett, C. & Salzberg, S. L. Graph-based genome alignment and genotyping with HISAT2 and HISAT-genotype. *Nat Biotechnol* **37**, 907-915 (2019).
17 Bray, N. L., Pimentel, H., Melsted, P. & Pachter, L. Near-optimal probabilistic RNA-seq quantification. *Nat Biotechnol* **34**, 525-527 (2016).
18 Herberg, S., Gert, K. R., Schleiffer, A. & Pauli, A. The Ly6/uPAR protein Bouncer is necessary and sufficient for species-specific fertilization. *Science* **361**, 1029-1033 (2018).
19 Cabrera-Quio, L. E., Schleiffer, A., Mechtler, K. & Pauli, A. Zebrafish Ski7 tunes RNA levels during the oocyte-to-embryo transition. *PLoS Genet* **17**, e1009390 (2021).
20 Wilson, L. D. *et al.* Fertilization in C. elegans requires an intact C-terminal RING finger in sperm protein SPE-42. *BMC Dev Biol* **11**, 10 (2011).
