# Supplementary material for: Sperm membrane proteins DCST1 and DCST2 are required for sperm-egg interaction in mice and fish
Source: Commun Biol. 2022 Apr 7;5:332. doi: 10.1038/s42003-022-03289-w (PMC8989947; doi:10.1038/s42003-022-03289-w)
Supplement: Supplementary file 3 — Description of Additional Supplementary Files [file 42003_2022_3289_MOESM3_ESM.pdf]

Description of Additional Supplementary Files

File name: Movie S1.
Description: Egg observation after IVF using Dcst1 KO sperm.

File name: Movie S2.
Description: Egg observation after IVF using Dcst2 KO sperm.

File name: Movie S3.
Description: Wild-type sperm approach to micropyle. Wild-type sperm stained with Mitotracker Deep-Red was added to wild-type eggs and images were acquired following sperm addition.

File name: Movie S4.
Description: Dcst2 mutant sperm approach to micropyle. Dcst2 mutant sperm stained with Mitotracker Deep-Red was added to wild-type eggs and images were acquired following sperm addition.

File name: Movie S5.
Description: Dcst2 mutant sperm are unable to stably bind to wild-type eggs. Time lapse of sperm binding assay with wild-type (left) and dcst2-/- (right) sperm stained with Mitotracker Deep Red and wild-type eggs. After 2 minutes following sperm addition, wild-type sperm are stably bound to the oolemma while dcst2-/- mutant sperm are unable to bind.

File name: Supplementary Data 1-10
Description:
Supplementary Data 1. Median-normalized level of *Dcst1* and *Dcst2* mRNA expression during mouse spermatogenesis. Each value of the median-normalized level in Figure 1B was shown. Ud Sg, undifferentiated spermatogonia; A1-A2 Sg, A1-A2 differentiating spermatogonia; A3-B Sg, A3-A4-In-B differentiating spermatogonia; Prele Sc, preleptotene spermatocytes; Le/Zy Sc, leptotene/zygotene spermatocytes; Pa Sc, pachytene spermatocytes; Di/Se Sc, diplotene/secondary spermatocytes; Early St, early round spermatids; Mid St, mid round spermatids; Late St, late round spermatids; SC, Sertoli cells.

Supplementary Data 2. Male fecundity. The pups/plug for each mating pair in Figure 1C was shown.

Supplementary Data 3. Sperm fertilizing ability using cumulus-intact eggs *in vitro*. The fertilization rates in Figure 1E were shown.

Supplementary Data 4. Sperm fertilizing ability using ZP-free eggs *in vitro*. The fertilization rates in Figure 1F were shown.

Supplementary Data 5. Fusion ability. The number of fused sperm and total number of observed eggs (see Figure 2F) were shown.

Supplementary Data 6. Rescue of male fertility. The pups/plug of *Dcst1*$^{d1/d1}$ males with *Dcst1*-3xHA Tg insertion and *Dcst2*$^{d25/d25}$ males with *Dcst2*-3xHA Tg insertion was shown (see Figure 3A).

Supplementary Data 7. The number of HEK293T cells bound to eggs. The adhesion ability of HEK293T cells overexpressing *Dcst1/2* and *Izumo1* to oocyte membrane was examined (see Figure 4C).

Supplementary Data 8. Fertilization rates of *dcst1*$^{-/-}$, *dcst2*$^{-/-}$, double mutant as well as wild-type zebrafish. The fertilization rates are provided per clutch of embryos; each clutch is derived from individual matings (see Figure 5A).

Supplementary Data 9. Quantification of zebrafish Dcst2 protein levels relative to Tubulin protein levels by Western Blotting. The Dcst2 band intensity was normalized to Tubulin band intensity and divided by the normalized wild-type Dcst2 band intensity (see Figure 5C).

Supplementary Data 10. Quantification of zebrafish wild-type or *dcst2*$^{-/-}$ sperm to wild-type activated eggs. Binding of sperm was assessed by counting the number of stably bound sperm in a 1-minute time window (see Figure 5F).
