# Supplementary material for: Sperm membrane proteins DCST1 and DCST2 are required for sperm-egg interaction in mice and fish
Source: Commun Biol. 2022 Apr 7;5:332. doi: 10.1038/s42003-022-03289-w (PMC8989947; doi:10.1038/s42003-022-03289-w)
Supplement: Supplementary file 10 — Reporting Summary [file 42003_2022_3289_MOESM10_ESM.pdf]

nature portfolio

Corresponding author(s): Pauli Andrea and Masahito Ikawa

Last updated by author(s): Jan 17, 2022

Reporting Summary

Nature Portfolio wishes to improve the reproducibility of the work that we publish. This form provides structure for consistency and transparency in reporting. For further information on Nature Portfolio policies, see our Editorial Policies and the Editorial Policy Checklist.

Statistics

For all statistical analyses, confirm that the following items are present in the figure legend, table legend, main text, or Methods section.

n/a	Confirmed	
☐	☒	The exact sample size (*n*) for each experimental group/condition, given as a discrete number and unit of measurement
☐	☒	A statement on whether measurements were taken from distinct samples or whether the same sample was measured repeatedly
☐	☒	The statistical test(s) used AND whether they are one- or two-sided<br>*Only common tests should be described solely by name; describe more complex techniques in the Methods section.*
☒	☐	A description of all covariates tested
☐	☒	A description of any assumptions or corrections, such as tests of normality and adjustment for multiple comparisons
☐	☒	A full description of the statistical parameters including central tendency (e.g. means) or other basic estimates (e.g. regression coefficient) AND variation (e.g. standard deviation) or associated estimates of uncertainty (e.g. confidence intervals)
☐	☒	For null hypothesis testing, the test statistic (e.g. *F*, *t*, *r*) with confidence intervals, effect sizes, degrees of freedom and *P* value noted<br>*Give P values as exact values whenever suitable.*
☒	☐	For Bayesian analysis, information on the choice of priors and Markov chain Monte Carlo settings
☒	☐	For hierarchical and complex designs, identification of the appropriate level for tests and full reporting of outcomes
☒	☐	Estimates of effect sizes (e.g. Cohen's *d*, Pearson's *r*), indicating how they were calculated

Our web collection on statistics for biologists contains articles on many of the points above.

Software and code

Policy information about availability of computer code

Data collection	No software was used for data collection.
Data analysis	For mouse data, statistical analyses were performed using Excel software with the add-in software Statcel3 (OMS, Saitama, Japan) (Fig. 2D, Fig. 4C, Fig. S6, S7B, and S8B). For zebrafish data, statistical analyses were performed in GraphPad Prism 7 software (Fig. 5A, 5C, and 5F).

For manuscripts utilizing custom algorithms or software that are central to the research but not yet described in published literature, software must be made available to editors and reviewers. We strongly encourage code deposition in a community repository (e.g. GitHub). See the Nature Portfolio guidelines for submitting code & software for further information.

Data

Policy information about availability of data

All manuscripts must include a data availability statement. This statement should provide the following information, where applicable:

- Accession codes, unique identifiers, or web links for publicly available datasets
- A description of any restrictions on data availability
- For clinical datasets or third party data, please ensure that the statement adheres to our policy

The authors declare that the data that support the findings of this study are available from the corresponding author upon request. RNA-seq data reported here (zebrafish adult tissues) were deposited at the Gene Expression Omnibus (GEO) and are available under GEO acquisition number GSE171906

Field-specific reporting

Please select the one below that is the best fit for your research. If you are not sure, read the appropriate sections before making your selection.

☒ Life sciences ☐ Behavioural & social sciences ☐ Ecological, evolutionary & environmental sciences

For a reference copy of the document with all sections, see nature.com/documents/nr-reporting-summary-flat.pdf

Life sciences study design

All studies must disclose on these points even when the disclosure is negative.

Sample size	Sample sizes were determined based on published studies in the field or our previous experiences. No statistics was used to predetermine the sample size.
Data exclusions	No samples or animals were excluded. Also, the criteria were not pre-established in experiments.
Replication	Experiments were performed using at least two biological replicas.
Randomization	Samples are defined by their unique genotypes. Therefore, no sample randomization was performed.
Blinding	The investigators were not blinded for group allocation.

Reporting for specific materials, systems and methods

We require information from authors about some types of materials, experimental systems and methods used in many studies. Here, indicate whether each material, system or method listed is relevant to your study. If you are not sure if a list item applies to your research, read the appropriate section before selecting a response.

Materials & experimental systems

n/a	Involved in the study	
☐	☒	Antibodies
☐	☒	Eukaryotic cell lines
☒	☐	Palaeontology and archaeology
☐	☒	Animals and other organisms
☒	☐	Human research participants
☒	☐	Clinical data
☒	☐	Dual use research of concern

Methods

n/a	Involved in the study	
☒	☐	ChIP-seq
☒	☐	Flow cytometry
☒	☐	MRI-based neuroimaging

Antibodies

Antibodies used	Rat monoclonal antibodies against mouse IZUMO1 (KS64-125) and mouse SLC2A3 (KS64-10) were generated by our laboratory as described previously46,47. The mouse monoclonal antibody against 1D4-tag was generated using a hybridoma cell line as a gift from Robert Molday, Ophthalmology and Visual Sciences, Centre for Macular Research, University of British Columbia, Vancouver, British Columbia, Canada48. Mouse monoclonal antibodies against the HA and FLAG tags were purchased from MBL (M180-3) and Sigma (F3165). The Alexa Fluor 488-conjugated Lectin PNA from Arachis hypogaea (peanut) was purchased from Thermo Fisher Scientific (L21409).<br>To generate mouse monoclonal antibodies against zebrafish Dcst1 and Dcst2, recombinant zebrafish Dcst1 (amino acids 590-675) and Dcst2 (amino acids 574-709) proteins were expressed in E. coli BL21(DE3) and purified by the VBCF Protein Technologies Facility. Each recombinant protein was injected into 3 mice, and monoclonal antibodies were generated by the Max Perutz Labs Monoclonal Antibody Facility according to standard procedures.<br>Horseradish peroxidase (HRP)-conjugated goat anti-mouse immunoglobulins (IgGs) (115-036-062) and HRP-conjugated goat anti-rat IgGs (112-035-167) were purchased from Jackson ImmunoResearch Laboratories. Fluorophore-conjugated secondary antibodies, goat anti-mouse IgG Alexa Fluor 488 (A11001), goat anti-mouse IgG Alexa Fluor 546 (A11018), goat anti-mouse IgG Alexa Fluor 594 (A11005), and goat anti-rat IgG Alexa Fluor 488 (A11006) were purchased from Thermo Fisher Scientific.
Validation	The mouse monoclonal antibody against zebrafish Dcst1 and Dcst2 was validated by western blot analysis and immunohistochemistry. All other antibodies were validated by previous papers and manufacturers.

Eukaryotic cell lines

Policy information about cell lines

Cell line source(s)	HEK293T cell line was gifted from Dr. Verma Lab (The Salk Institute).

Authentication	Cell lines were authenticated based on their morphology and growth.
Mycoplasma contamination	Mycoplasma contamination was examined using TaKaRa PCR Mycoplasma Detection Set (Takara; 6601). HEK293 was tested and nagative for mycoplasma contamination.
Commonly misidentified lines (See ICLAC register)	No commonly misidentified lines were used.

Animals and other organisms

Policy information about studies involving animals; ARRIVE guidelines recommended for reporting animal research

Laboratory animals	B6D2F1, C57BL/6J, and ICR mice were purchased from Japan SLC and CLEA Japan. Mice were acclimated to 12-h-light/12-h-dark cycle. Zebrafish (Danio rerio) were raised according to standard protocols (28°C water temperature; 14/10-hour light/dark cycle). TLAB zebrafish served as wild-type zebrafish for all experiments, and were generated by crossing zebrafish AB stocks with natural variant TL (Tüpfel longfin) stocks.
Wild animals	This study did not involve wild animals.
Field-collected samples	This study did not involve field-collected samples.
Ethics oversight	All mouse experiments were approved by the Animal Care and Use Committee of the Research Institute for Microbial Diseases, Osaka University, Japan (#Biken-AP-H30-01) and the Animal Care and Use Committee of Kumamoto University (ID: A2021-035). All fish experiments were conducted according to Austrian and European guidelines for animal research and approved by the local Austrian authorities (Amt der Wiener Landesregierung, Magistratsabteilung 58 - Wasserrecht; animal protocols GZ: 342445/2016/12 and MA 58-221180-2021-16).

Note that full information on the approval of the study protocol must also be provided in the manuscript.
